# Ageing in a Changing Climate: Risks, Adaptations, and Health Impacts

**DOI:** 10.1093/geroni/igaf122.790

**Published:** 2025-12-31

**Authors:** Paola Zaninotto, Liat Ayalon

## Abstract

Older adults are disproportionately vulnerable to climate change, yet research and policy often overlook their specific risks and adaptive strategies. This symposium examines the intersection of ageing, environmental hazards, and health, shedding light on the lived experiences and physiological impacts of climate change on older populations. We begin by exploring how older adults navigate disaster recovery in hazardous environments, using Hurricane Ian as a case study to highlight social infrastructure needs and barriers to ageing in place. Next, we examine the complex relationship between thermal perception, acceptance, and physical symptoms, offering insights into heat adaptation behaviours among older individuals. A further presentation addresses the respiratory health risks associated with domestic solid fuel use, revealing its dual impact on individual health and climate change. Lastly, we present findings on the effects of temperature on kidney function, providing further evidence of climate-related health risks for older adults. Together, these studies emphasise the urgent need for policies and interventions that enhance resilience and mitigate health risks in an ageing population. By integrating perspectives from different disciplines, this symposium offers a critical dialogue on the challenges and opportunities of ageing in a warming world.

